# “Asymmetric scalloping of the regenerate”: a radiological sign of pseudoaneurysm in distraction osteogenesis

**DOI:** 10.1007/s11751-011-0121-4

**Published:** 2011-11-18

**Authors:** J. Fagg, A. Gulihar, J. A. Fernandes

**Affiliations:** 1 Registrar Trauma and Orthopaedic Surgery, Barnsley District General Hospital, Barnsley, South Yorkshire UK; 2 Speciality Registrar Trauma and Orthopaedic Surgery, Sheffield Children’s Hospital, Western Bank, Sheffield, S10 2TH UK; 3 Consultant Paediatric Orthopaedic Surgeon, Sheffield Children’s Hospital, Western Bank, Sheffield, S10 2TH UK

**Keywords:** Asymmetric scalloping, Regenerate, Pseudoaneurysm, External fixator, Distraction osteogenesis

## Abstract

Pseudoaneurysm formation is an uncommon but well-recognised and important complication in limb reconstruction surgery. Postoperative diagnosis is usually clinical or an incidental finding. We present an 11-year-old girl, who underwent two-stage limb lengthening with a circular fixator, for a previously treated pseudoarthrosis of the tibia. During the lengthening plan, a concave defect was noted on one side of the regenerate, which was found to be due to extrinsic compression by a pseudoaneurysm. Normal regenerate formation was seen after selective embolisation of the pseudoaneurysm. This concave appearance on one side of the regenerate has previously been described secondary to a difference in stability on the two sides of the osteotomy, when a monolateral fixator is used, but not due to extrinsic compression by a pseudoaneurysm. The authors propose that this radiographic appearance of “asymmetrical scalloping” on one side of the regenerate may represent a radiological sign of a pseudoaneurysm formation and should provoke investigation for the same.

## Introduction

Distraction osteogenesis is an established technique for limb lengthening and deformity correction, for both congenital and acquired deformities [1–3]. Fixation and distraction devices include internal [4, 5] and external fixators [1, 3]. Vascular injuries associated with osteotomies and with external fixation devices are well documented, including the formation of a pseudoaneurysm [6–9]. Pseudoaneurysms present in a number of different ways, ranging from acute, life-threatening episodes to asymptomatic incidental findings [9, 10]. We present a case of an asymptomatic pseudoaneurysm that was noticed during distraction, where the diagnosis was suspected based on the plain radiographic appearance of the regenerate.

## Case report

A 15-year-old girl underwent a proximal tibial osteotomy and application of Ilizarov frame for second stage lengthening of her right leg. Her primary diagnosis was of congenital pseudoarthrosis of the right tibia successfully treated previously with a vascularised fibular graft. She had also undergone a first stage lengthening of about 3 cm at the age of 12 years. A two-stage reconstruction had been offered at that stage to avoid traction injury to the vascular anastomosis.

**Fig. 1:**
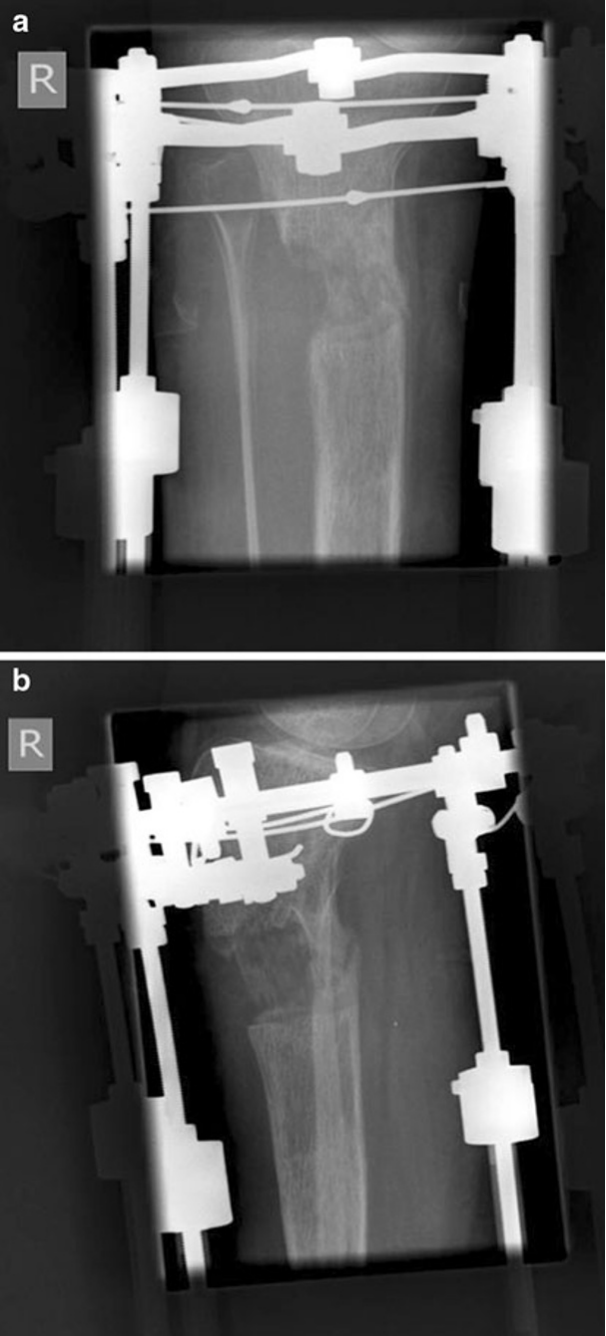
**a** Anteroposterior and **b** lateral radiographs of the proximal tibia 4 months into distraction osteogenesis showing “asymmetric scalloping” of the posterolateral aspect of the regenerate

**Fig. 2:**
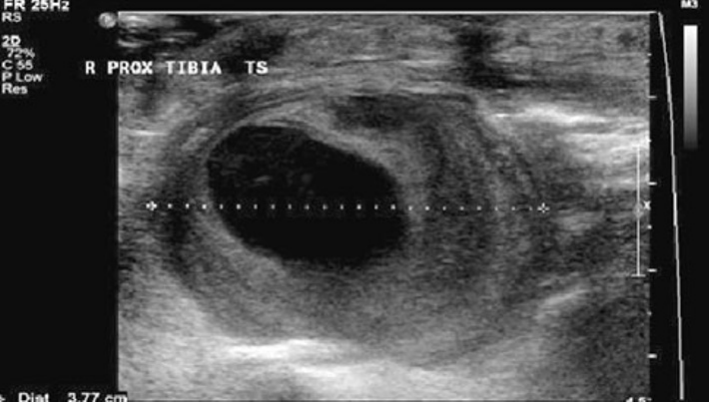
Ultrasound scan around the pin sites revealing a large mass with turbulent flow, suspicious of a pseudoaneurysm

A venous ooze was observed from the proximal metaphysis during surgery, but this settled with osteotomy compression. A standard limb lengthening protocol was followed postoperatively. The patient had a minor fall 4 months postoperatively and noticed some bleeding from the pin sites, which settled spontaneously. A plain radiograph at this stage showed delayed asymmetric regenerate on the posterolateral aspect of the callotasis site, giving an appearance of “scalloping” (Fig. 1). This appearance raised the suspicion of a local pressure effect and an ultrasound scan was organised. This demonstrated the presence of a large, 3.8 × 3.8 × 2.9 cm mass (Fig. 2) with turbulent flow adjacent to one of the fixator wires. The patient was referred to the vascular radiology department where an angiogram was performed confirming the presence of a pseudoaneurysm arising from the anterior tibial artery. Due to adequate alternative perfusion of the foot, coil embolisation of the anterior tibial artery was performed without further complications and with full cessation of flow in the pseudoaneurysm.

**Fig. 3:**
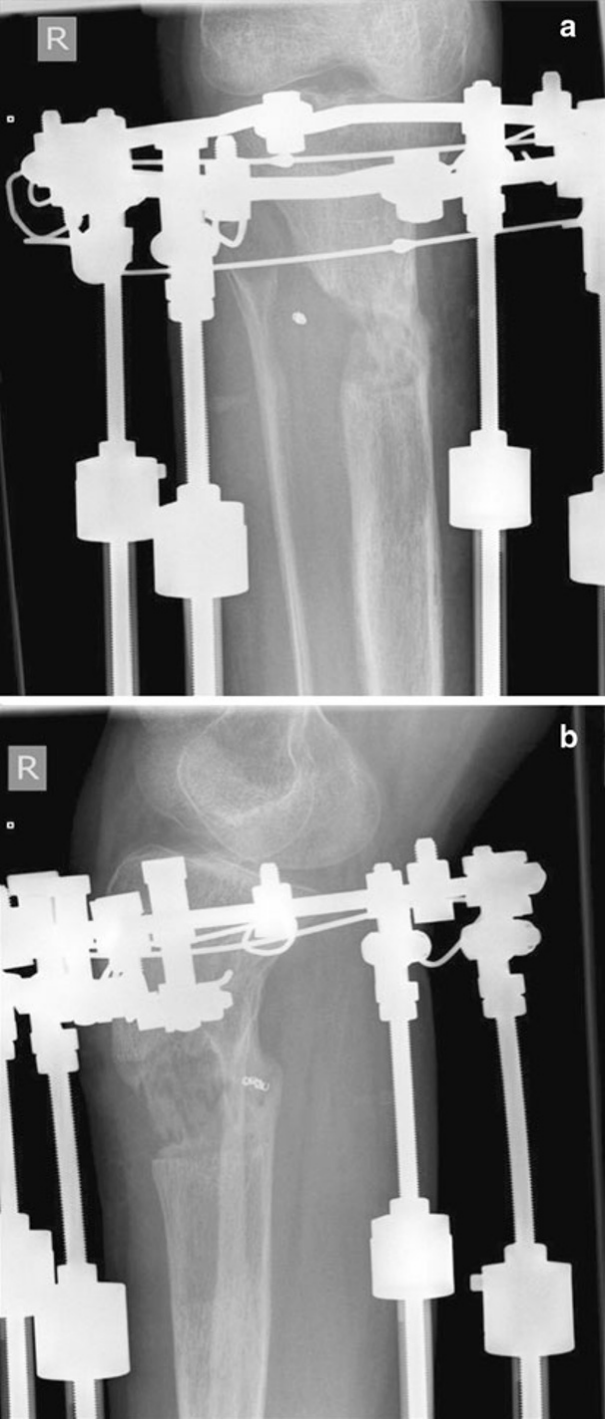
**a** Anteroposterior and **b** lateral radiograph of the tibia 1-month after angiographic coil embolisation of the pseudoaneurysm arising from the anterior tibial artery. A reduction in the concavity of the regenerate was noted. The embolisation coil can be seen at the site of the pseudoaneurysm

**Fig. 4:**
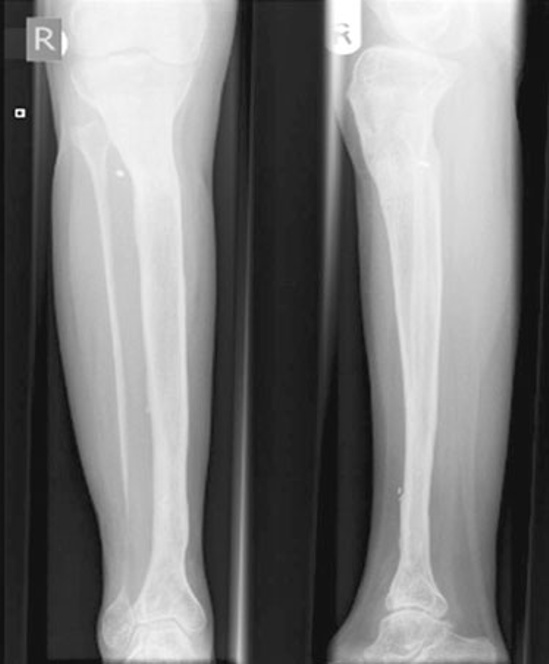
Plain radiographs at 1-year follow-up showing a fully healed corticotomy site with filling-in of the previous scalloping

One-month after the procedure, the regenerate was seen to consolidate further with a decrease in the concavity of the scalloping (Fig. 3). The fixator was removed 8 months postoperatively. Further radiographs 1-year postoperatively showed a fully healed corticotomy site with filling-in of the defect (Fig. 4).

## Discussion

Vascular injuries are well-recognised complications of external fixators [6–9]. Several reports of pseudoaneurysms have been described, more common in the lower limb. Dhal et al. [6] described five such cases and outlined the pathogenesis of pseudoaneurysm formation. Traumatic pseudoaneurysms form after arterial wall injury leads to bleeding into the soft tissues around the vessel. The uninjured portion of the arterial wall prevents the vessel from contracting leading to extravasation of blood and haematoma formation. This haematoma undergoes organisation and develops a fibrous capsule with central liquefaction creating a secular swelling directly communicating with the arterial lumen [5].

If untreated, pseudoaneurysms can result in rupture and haemorrhage, occlusion, distal embolisation, infection and local pressure effects on surrounding structures. They can be treated in the early stages of the development by compression, or in late stages, by ligation, surgical excision, covered stent exclusion or angiographic embolisation [6, 11]. Aneurysms of one of the three leg arteries are best treated by selective angiographic embolisation, commonly with metal coils, provided that the other vessels are healthy enough to provide good collateral flow.

The above case is unusual in that the pseudoaneurysm was completely asymptomatic, but had a clear pressure effect on the regenerate bone, which was picked up by radiographic images. The aetiology of this pseudoaneurysm is difficult to ascertain as the anatomical location of the aneurysm may suggest arterial injury at the time of the osteotomy, but the ultrasound appearances suggested a neck related to a wire site. Previous surgery being performed in the same region probably increases the chance of arterial injury. This is due to the presence of scar tissue preventing normal vessel mobility, already limited in this case by the tethering of the anterior tibial artery to the interosseous membrane. This makes intraoperative injury more likely and predisposes to further arterial injury during distraction.

Li et al. [12] described five different shapes of regenerates: fusiform, cylindrical, concave, lateral and central. They felt that a “concave” regenerate on both sides was due to poor bony response or a vigorous lengthening programme. However, a one-sided defect, also called as “lateral”, was thought to be due to differences in circulation, soft tissue cover or stability on the two sides of the osteotomy. Donnan et al. [13] described this shape as “opposite”, stating that this shape is only seen on the side opposite to a monolateral fixator, implying that this appearance was related to stability. This problem is not seen with circular frames, which act by beam loading. We believe that this appearance can also be due to extrinsic compression, as seen in our case.

This appearance of a concave regenerate defect on one side as a result of extrinsic compression has previously not been reported in the current literature. We propose that this “asymmetric scalloping” of the regenerate, in the presence of a stable construct, should be considered as a radiological sign of pseudoaneurysm formation and when present, should spark a low threshold for imaging the vasculature surrounding the pin sites.
